# Supplementary material for: Acute melanization of silkworm hemolymph by peptidoglycans of the human commensal bacterium Cutibacterium acnes
Source: PLoS One. 2022 Sep 26;17(9):e0271420. doi: 10.1371/journal.pone.0271420 (PMC9512201; doi:10.1371/journal.pone.0271420)
Supplement: S1 File — (DOCX) [file pone.0271420.s001.docx]

**Acute melanization of silkworm hemolymph by peptidoglycans of the human commensal bacterium *Cutibacterium acnes***

**Yasuhiko Matsumoto^1*^, Eri Sato^1^, and Takashi Sugita^1^**

^1^Department of Microbiology, Meiji Pharmaceutical University, 2-522-1, Noshio, Kiyose, Tokyo 204-8588, Japan.

*Corresponding author

E-mail: ymatsumoto@my-pharm.ac.jp (YM)


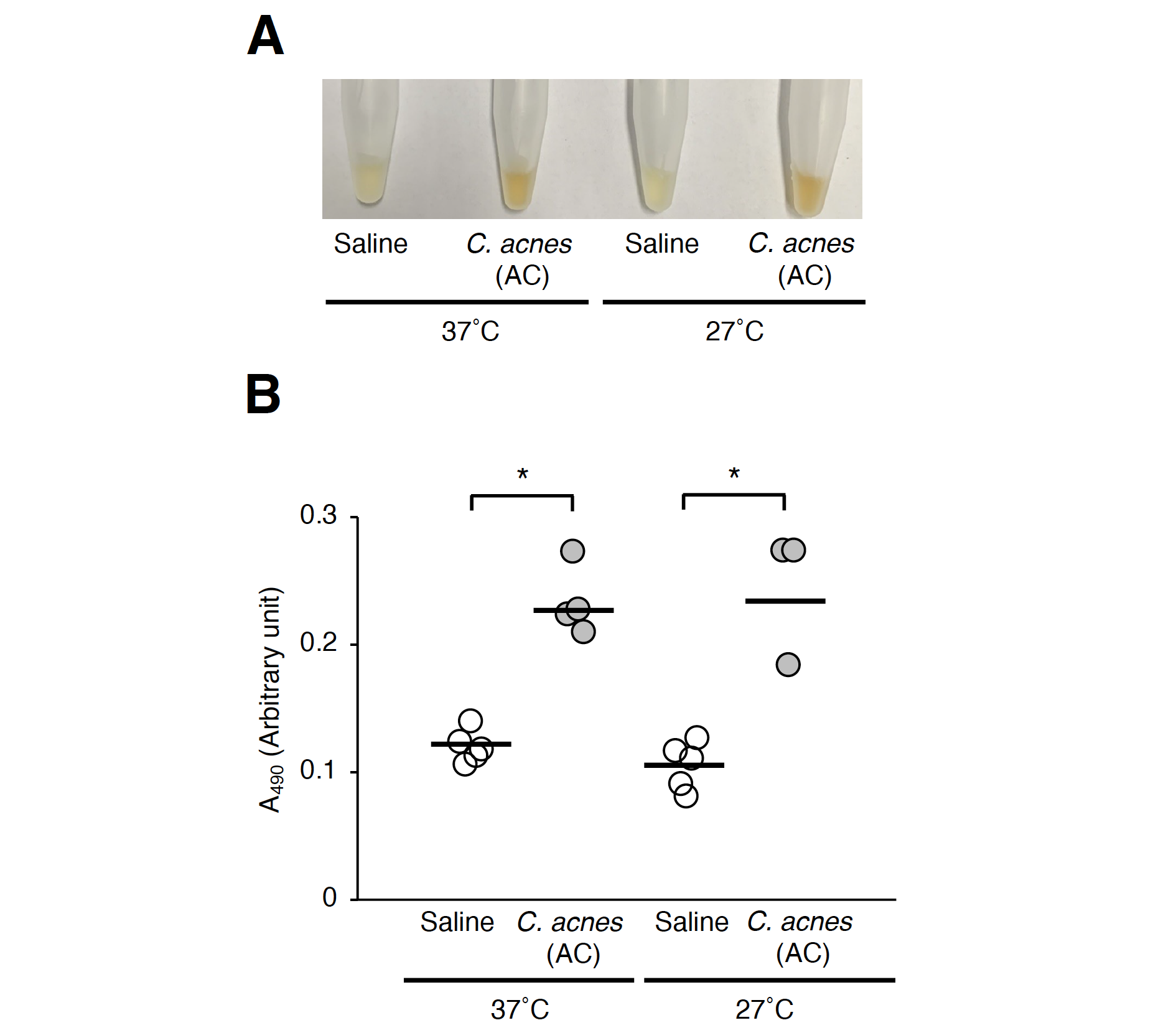


**Supplementary Fig. S1 Effect of rearing temperature on the silkworm hemolymph melanization by *C. acnes* (AC).**

(**A**, **B**) Silkworms were injected with 50 µl of saline (Saline) or the autoclaved *C. acnes* cell suspension (*C. acnes* [AC]: OD_600_ = 1.0). After incubation for 3 h at 37˚C, the silkworm hemolymph was collected. Photographs of the silkworm hemolymph were taken (**A**) and absorbance at 490 nm (A_490_) (**B**) was measured. n = 3-5/group. Statistically significant differences between groups were evaluated using the Tukey test. **P* < 0.05. The arbitrary unit was defined as the absorbance at 490 nm of a sample of silkworm hemolymph (50 µl) mixed with saline (50 µl).


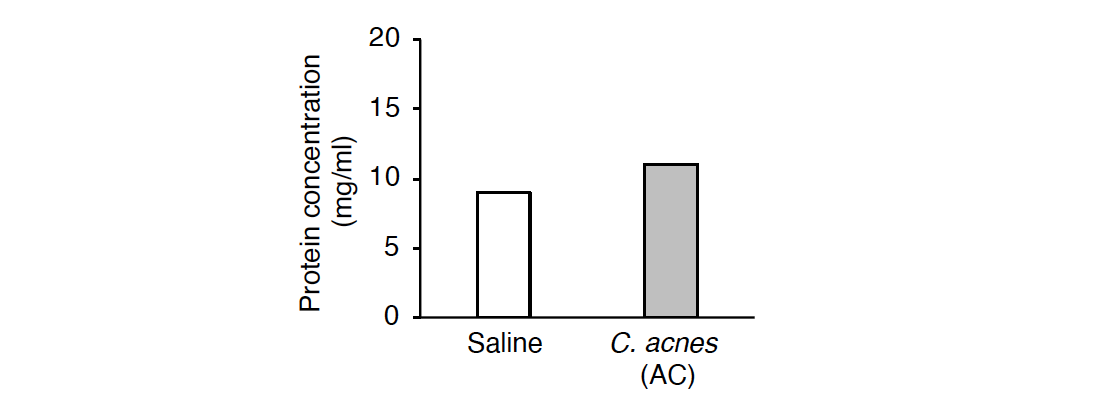


**Supplementary Fig. S2 Effect of melanization after injection of *C. acnes* (AC) on the protein concentration of silkworm hemolymph.**

Silkworms were injected with 50 µl of saline (Saline) or the autoclaved *C. acnes* cell suspension (*C. acnes* [AC]: OD_600_ = 1.0). After incubation for 3 h at 37˚C, the silkworm hemolymph was collected. The protein concentration of silkworm hemolymph was measured on Bradford method. Bradford Dye Reagent was purchased from Takara Bio Inc. (Shiga, Japan).


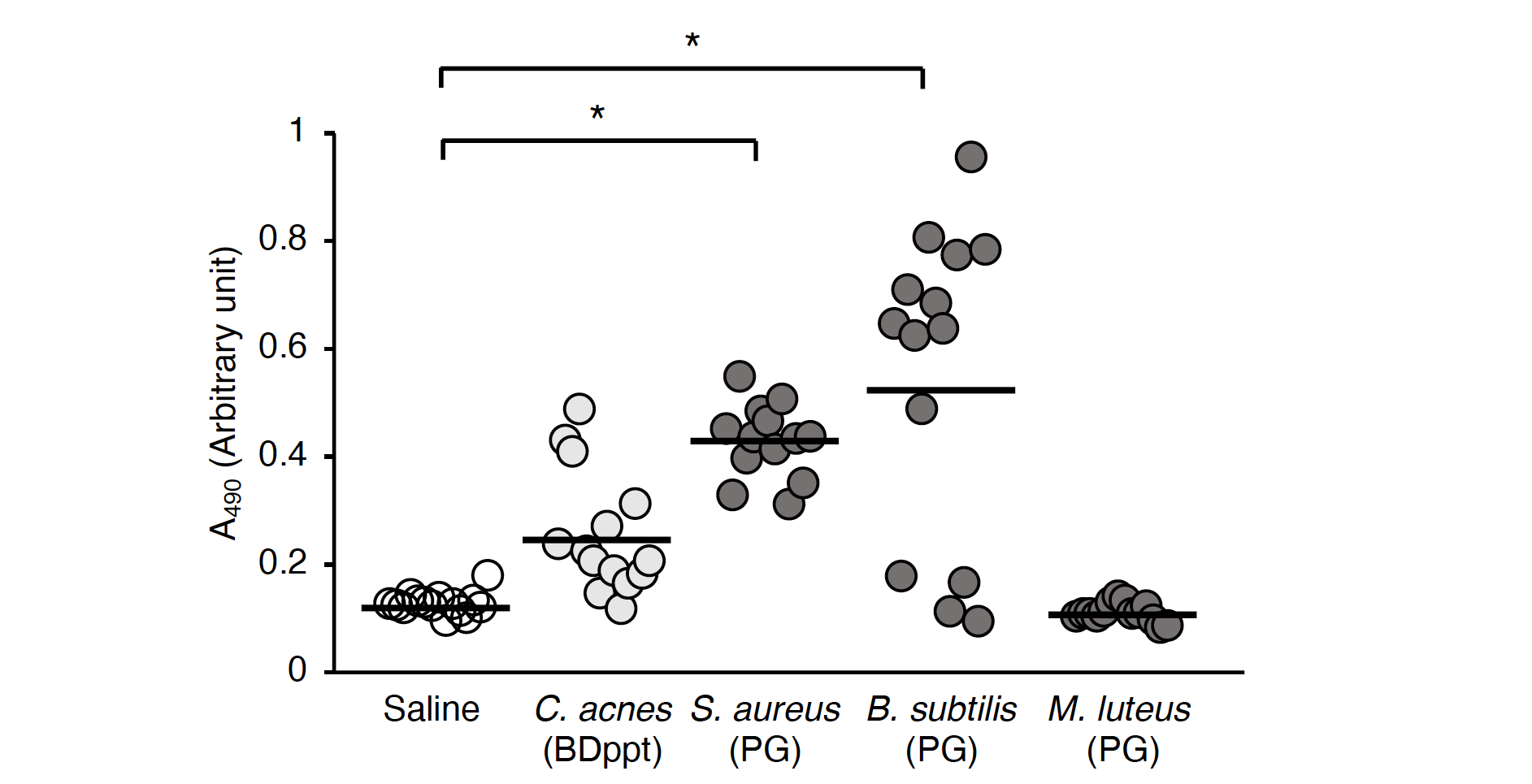


**Supplementary Fig. S3 Melanization-inducing activities of bacterial peptidoglycans.**

Silkworms were injected with 50 µl of saline (Saline), the *C. acnes* BDppt fraction (BDppt), *S. aureus*, *B. subtilis*, or *M. luteus* peptidoglycans (PG)(OD_600_ = 0.15). After incubation for 3 h at 37˚C, the silkworm hemolymph was collected. Absorbance at 490 nm (A_490_) was measured. The arbitrary unit was defined as the absorbance at 490 nm of a sample of silkworm hemolymph (50 µl) mixed with saline (50 µl). n = 13-15/group. Statistically significant differences between groups were evaluated using the Tukey-Kramer test. **P* < 0.05.
